# Biallelic loss-of-function *PTPN6* variants lead to inflammatory lung disease and hemolytic anemia

**DOI:** 10.1084/jem.20252379

**Published:** 2026-10-07

**Authors:** S. Farshid Moussavi-Harami, Matt S. Zinter, Marialetizia Motta, Luca Pannone, Paolo Calligari, Luca Mignini, Anne Slavotinek, Pierre-Marie Martin, Henry Sanchez, Lauren Sanchez, Iman Ragab, Clare L. Abram, Martin Broly, Yasmina Abi Ayad, Amina Serradj, Guilaine Boursier, Aida M. Bertoli-Avella, Mukunth Sadagopan, Nima Parvaneh, Sahar Sedighzadeh, Ebrahim Mirzaei, Mohammad Shahrooei, Amy P. Hsu, Steven M. Holland, Gianfranco Bocchinfuso, Lorenzo Stella, Simone Martinelli, Bruno Reversade, Marco Tartaglia, Clifford A. Lowell, Mark R. Looney, Alice Y. Chan

**Affiliations:** 1 Division of Pediatric Critical Care Medicine, Department of Pediatrics, University of California, San Francisco, San Francisco, CA, USA; 2 Molecular Genetics and Functional Genomics, Bambino Gesù Children’s Hospital, IRCCS, Rome, Italy; 3 Department of Oncology and Molecular Medicine, Istituto Superiore di Sanità, Rome, Italy; 4 Department of Chemical Science and Technologies, University of Rome Tor Vergata, Rome, Italy; 5 Division of Human Genetics, Department of Pediatrics, Cincinnati Children’s Hospital Medical Center, Cincinnati, OH, USA; 6 University of Cincinnati College of Medicine, Cincinnati, OH, USA; 7 Institute of Human Genetics, University of California, San Francisco, San Francisco, CA, USA; 8 Department of Pathology and Laboratory Medicine, University of California, San Francisco, San Francisco, CA, USA; 9 Division of Allergy, Immunology and Bone Marrow Transplantation, Department of Pediatrics, University of California, San Francisco, San Francisco, CA, USA; 10 Hematology Oncology Unit, Ain Shams University, Children’s Hospital, Pediatric Hospital, Cairo, Egypt; 11 Department of Laboratory Medicine, University of California, San Francisco, San Francisco, CA, USA; 12 Rare and Autoinflammatory Genetic Diseases Unit, Laboratory of Molecular Genetics and Rare Diseases R+3, CEREMAIA – Site unique de biologie, CHU Montpellier, Montpellier, France; 13 Faculty of Médicine University Oran1 Ahmed Benbella, Oran, Algeria; 14 Department of Molecular Genetics and Cytogenomics, Reference Center for Autoinflammatory Diseases and Amyloidosis (CeRéMAIA), Institute for Regenerative Medicine and Biotherapy, INSERM U1183, CHU Montpellier, University Montpellier, Montpellier, France; 15 Centogene AG, Rostock, Germany; 16 Division of Allergy and Clinical Immunology, Department of Pediatrics, Children’s Medical Center, Tehran University of Medical Sciences, Tehran, Iran; 17 Dr. Shahrooei Lab, Tehran, Iran; 18 Department of Medical Genetics, School of Medicine, Iran University of Medical Sciences, Tehran, Iran; 19 Laboratory of Clinical Immunology and Microbiology, National Institute of Allergy and Infectious Diseases, National Institutes of Health, Bethesda, MD, USA; 20 National Institute of Allergy and Infectious Diseases, National Institutes of Health, Bethesda, MD, USA; 21 Laboratory of Human Genetics & Therapeutics, Biological and Environmental Sciences and Engineering Division (BESE), King Abdullah University of Science and Technology (KAUST), Thuwal, Kingdom of Saudi Arabia; 22 Genome Institute of Singapore (GIS), Agency for Science, Technology and Research A*STAR, Singapore; 23 Medical Genetics Department, Koç University School of Medicine (KUSOM), Istanbul, Turkiye; 24 National University of Singapore (NUS), Cardiovascular-Metabolic Disease Translational Research Program (CVMD-TRP), Yong Loo Lin School of Medicine, NUS, Singapore; 25 Division of Pulmonary, Critical Care, Allergy and Sleep Medicine, Department of Medicine, University of California, San Francisco, San Francisco, CA, USA

## Abstract

Severe inflammatory lung disease, including acute respiratory distress syndrome (ARDS), is life-threatening with diverse causes and few targeted therapies. Inborn errors of immunity (IEIs) underlie a subset of cases. We describe a novel IEI caused by biallelic loss-of-function variants in *PTPN6*, encoding the immunoregulatory phosphatase SHP1. Seven children from five families developed early-onset anemia and severe inflammatory lung disease, at times presenting as ARDS. The causative variants were shown to destabilize SHP1 and abolish its phosphatase activity. These findings expand the genetic landscape of inflammatory lung disease and suggest SHP1 activation as a potential therapeutic strategy for immune-mediated pulmonary pathology.

## Teaser

Moussavi-Harami et al. report that biallelic loss-of-function variants in PTPN6, encoding the immune-regulatory phosphatase SHP1, cause anemia and fatal lung hyperinflammation in families from diverse ethnic backgrounds.

## Introduction

Severe inflammatory lung diseases, such as acute respiratory distress syndrome (ARDS), contribute significantly to morbidity and mortality. Despite medical advances, outcomes in ARDS remain largely unchanged, and survivors face risks of pulmonary fibrosis and chronic respiratory failure (Wick et al., 2024). Recent therapeutic successes in COVID-19–associated ARDS with immunomodulatory therapies underscore the role of host immune responses in ARDS pathogenesis (Horby et al., 2021, Recovery Collaborative Group, 2021; Selvaraj et al., 2022). Genetic studies in severe COVID-19 ARDS have identified pathogenic variants in type I IFN (Zhang et al., 2020) and Toll-like receptor signaling pathways as disease drivers (Matuozzo et al., 2023; Zhang et al., 2022). These findings highlight how inborn errors of immunity (IEIs) can reveal disease-driving pathways and may inform therapeutic strategies.

SHP1, encoded by *PTPN6*, is a protein tyrosine phosphatase (PTP) previously demonstrated to regulate inflammation and lung injury in preclinical mouse models of ARDS (Moussavi-Harami et al., 2024). It is expressed predominantly in hematopoietic cells, with lower levels in endothelial and epithelial cells. Notably, *PTPN6* is regulated by two distinct promoters, giving rise to a slightly longer isoform in non-hematopoietic cells compared with the hematopoietic-specific form. SHP1-deficient mice, termed motheaten, develop multiorgan immune-mediated disease, including lung inflammation and dermatitis, due to immune hyperactivation (Abram et al., 2013; Green and Shultz, 1975; Pao et al., 2007). The phosphatase regulates the intracellular signaling elicited by various cytokines and growth factors (e.g., GM-CSF, interleukins, and IFN) to modulate cell proliferation, differentiation, survival, and apoptosis in adaptive and innate immunity (Abram and Lowell, 2017; Garg et al., 2020; Hardin et al., 2006; Zhang et al., 2000). In B cells, SHP1 reduces responses to extracellular stimuli and contributes to immune tolerance through interactions with immunoreceptor tyrosine-based inhibitory motif (ITIM) containing co-receptors of the B cell receptor complex, including CD22, CD72, and PIR-B (Adachi et al., 2001; Lankester et al., 1995; Maeda et al., 1999). In T cells, SHP1 negatively regulates TCR signaling by dephosphorylating early signaling molecules, including Lck and ZAP-70, thereby raising the TCR activation threshold (Cuevas et al., 1999; Plas et al., 1996). Furthermore, loss of SHP1 leads to Th2- and Th17-skewed T cell hyperresponsiveness (Kamata et al., 2003; Mauldin et al., 2012). In myeloid cells, SHP1 acts as a central inhibitor of cell activity by regulating integrin outside-in signaling, cytokine-induced reactive oxygen species production, and phagocytosis (Kruger et al., 2000; Roach et al., 1998; Stadtmann et al., 2015). Cell-specific deletion of SHP1 in neutrophils and dendritic cells recapitulates some of the motheaten phenotype, underscoring the role of the protein in regulating innate immunity (Abram et al., 2013). In macrophages, SHP1 is recruited by SIRPα, leading to reduced STAT1, NF-κB, and MAPK signaling (Alblas et al., 2005; Shi et al., 2021). Furthermore, SHP1 inhibits phagocytosis of red blood cells by tissue-resident macrophages via the CD47-SIRPα signaling network (Okazawa et al., 2005).

While murine models of SHP1 loss have been widely utilized to study immune regulation, direct evidence linking SHP1 to human disease has remained limited. There have been two families with genetic variants in the *PTPN6* locus, presenting with differing clinical phenotypes: neutrophilic dermatosis or smoking-associated early-onset emphysema (Bossé et al., 2019; Nesterovitch et al., 2011; Parsapour et al., 2003). Notably, one of the two patients in the family with neutrophilic dermatosis died at 4 years due to pulmonary hypertension and diffuse vasculitis (Parsapour et al., 2003). Additionally, reduced SHP1 protein expression has been observed in peripheral blood mononuclear cells from patients with multiple sclerosis, and *PTPN6* mutations have been identified in various types of cancers (Christophi et al., 2008; Varone et al., 2020). However, these findings lack consistent genetic and phenotypic evidence to definitively link *PTPN6* to specific human diseases.

Here, we report seven patients from five families with biallelic loss-of-function (LoF) variants in *PTPN6*, who developed severe, often lethal, inflammatory lung disease and early-onset anemia. A broad spectrum of variants was identified, including missense substitutions, in-frame indels, and truncating changes that variably reduce the catalytic activity of SHP1, impair protein synthesis, or cause accelerated degradation. Our cases expand the IEI spectrum linked to severe inflammatory lung disease and suggest SHP1-related pathways as potential therapeutic targets.

## Results and discussion

### Clinical manifestations

**Figure 1.**
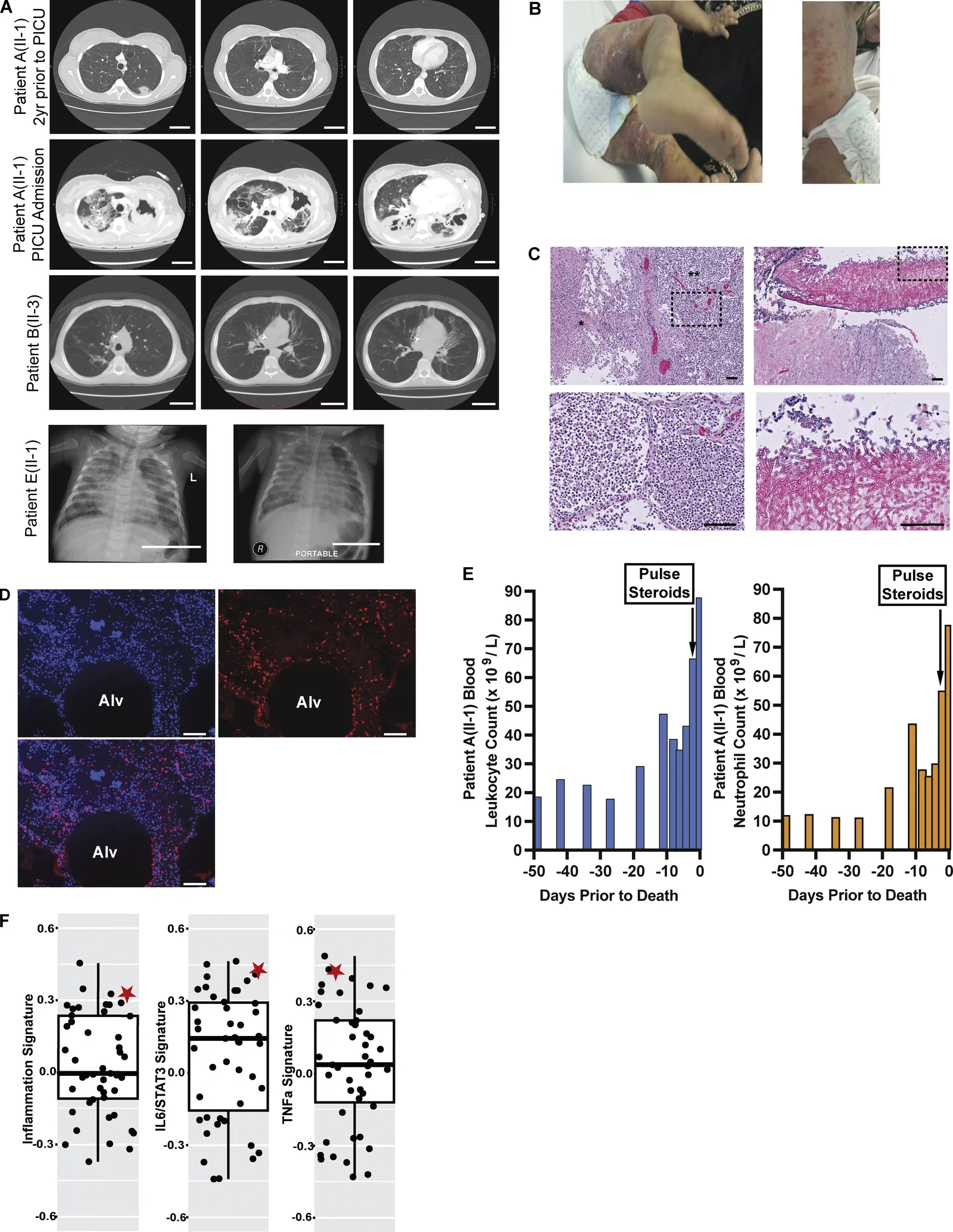
**Primary immune regulatory disorder leading to multiorgan dysfunction associated with *PTPN6* variants. (A)** Lung computed tomography of patients A(II-1) and B(II-3), indicating bronchiectasis, large cystic lesions in both patients, and focal lobar pneumonia in patient A(II-1). Chest x-ray of patient E(II-1) with ground-glass opacities. Scale bars = 5 cm. **(B)** Photograph of patient D(II-2) at 3 years of age, indicating severe dermatitis. **(C)** H&E tissue histology from patient A(II-1) indicating acute necrotizing pneumonia with a large abscess of the lower lobe of the right lung (left panels). The tissue section demonstrates acute necrotizing pneumonia (**) with a large abscess cavity measuring 11 × 6 cm (*) with a thin organizing fibrous wall in the middle (100×) (top left). Acute necrotizing pneumonia with a large abscess of the lower lobe of the right lung. Higher magnification view of acute necrotizing pneumonia with a large abscess of the lower lobe of the right lung with numerous intra-alveolar neutrophils and scattered foamy macrophages (200×) (bottom left). An intracavitary aspergilloma of the lower lobe of the left lung (right panels). The tissue section of an intracavitary lesion measuring 5.5 × 2.5 cm lined with *Aspergillus* species (100×) (top right). Higher magnification view of intracavitary aspergilloma of the lower lobe of the left lung. The tissue section shows dark blue-purple uniform, regularly septate hyphae with acute-angle branching representing viable *Aspergillus* species, while the underlying reddish-pink septate hyphae are nonviable fungus (400×) (bottom right). Dashed lines indicate the region of interest imaged at high magnification in a serial section. **(D)** Immunofluorescent staining of paraffin-embedded lung tissue from patient A(II-1) labeled by DAPI (DNA, blue) and MPO (neutrophils, red), indicating a neutrophilic predominance (Alv—alveolar space). Left panel: DAPI only; right panel: MPO only; bottom panel: DAPI plus MPO. Scale bars = 100 µm. **(E)** Total peripheral blood leukocyte and neutrophil count for patient A(II-1), indicating significant leukocytosis and neutrophilia prior to steroid administration and death. **(F)** Gene set enrichment scores were calculated for patient A(II-1) (indicated as a red star) and compared with a cohort of pediatric patients with septic shock.

**Figure 2.**
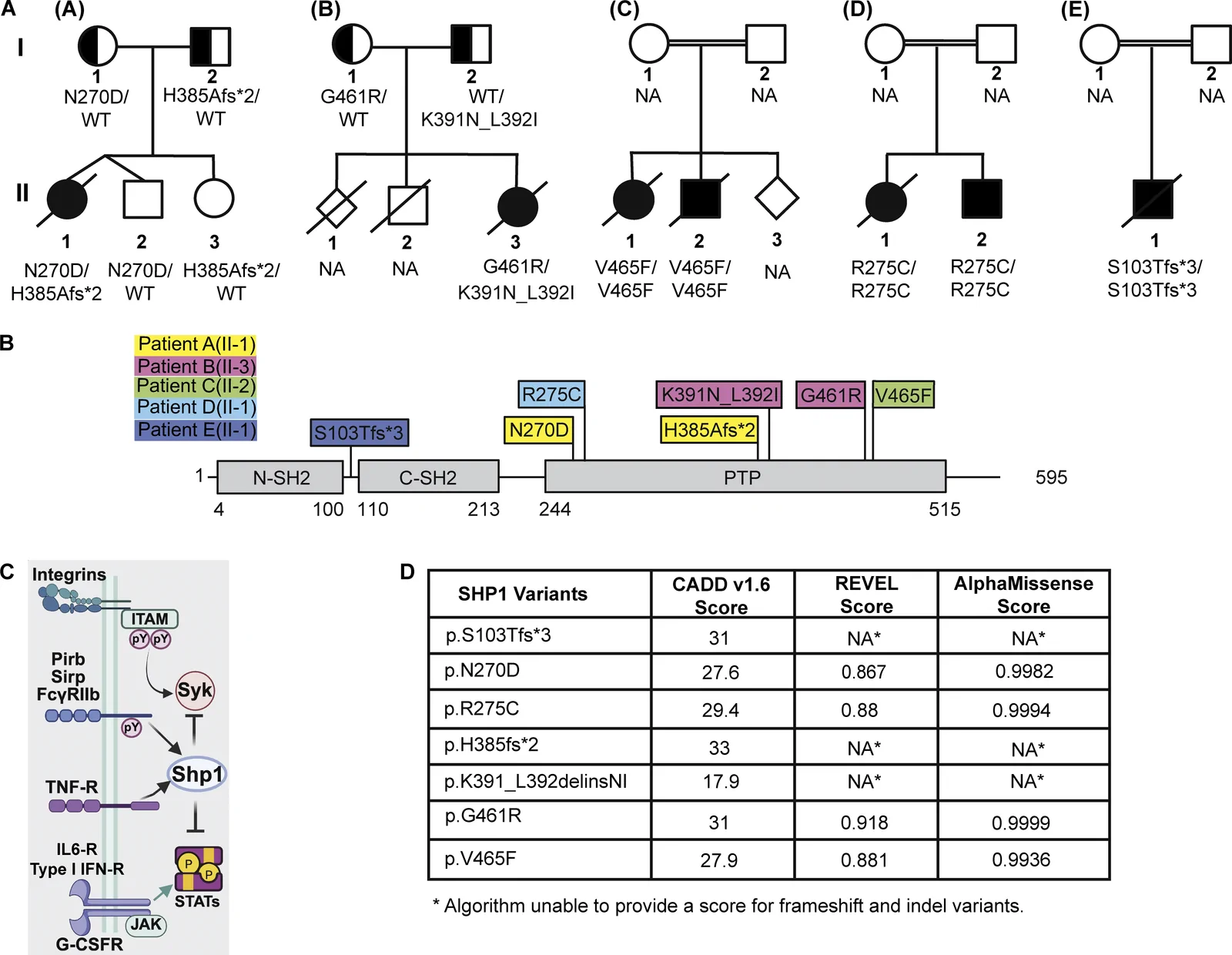
**Biallelic pathogenic variants of *PTPN6*, encoding SHP1, in patients with primary immune regulatory disorder. (A)** Pedigree of identified patients from five families (A–E) with associated *PTPN6* variants. **(B)** Diagram of *PTPN6* variants with one variant between the SH2 domains and six variants in the PTP domain of the protein. **(C)** Signaling pathways regulated by SHP1. **(D)** Computational prediction algorithms (CADD, REVEL, and AlphaMissense) of the SHP1 variants.

Seven children from five unrelated families were identified with infant-onset hemolytic anemia and severe inflammatory lung disease (Table 1, Fig. 1 and Fig. 2 A).

**Table 1.** Summary of clinical characteristics for the identified families

​	Family A	Family B	Family C		Family D		Family E
Patient ID	A(II-1)	B(II-3)	C(II-1)	C(II-2)	D(II-1)	D(II-2)	E(II-1)
Maternal allele	c.808A>G p.Asn270Asp (p.N270D)	c.1381G>C p.Gly461Arg (p.G461R)	c.1393G>T p.Val465Phe (p.V465F)	c.1393G>T p.Val465Phe (p.V465F)	c.823C>T p.Arg275Cys (p.R275C)	c.823C>T p.Arg275Cys (p.R275C)	c.292_305dup p.Ser103Thrfs*3 (p.S103Tfs*3)
Paternal allele	c.1151dupA p.His385Alafs*2 (p.H385AFs*2)	c.1173_1174delinsTA p.Lys391_Leu392delinsAsnIle (p.K391_L392delinsNI)	c.1393G>T p.Val465Phe (p.V465F)	c.1393G>T p.Val465Phe (p.V465F)	c.823C>T p.Arg275Cys (p.R275C)	c.823C>T p.Arg275Cys (p.R275C)	c.292_305dup p.Ser103Thrfs*3 (p.S103Tfs*3)
Biological sex	Female	Female	Female	Male	Female	Male	Male
Family history	Father with clubbing	First child died at birth Older brother died (8 yo) of hemolytic anemia, recurrent pneumonia infections (mycobacterial and fungal)	Consanguineous	Consanguineous	Consanguineous (parents first cousins)	Consanguineous (parents first cousins)	Consanguineous (parents first cousins)
Ancestry	African American	Mexican	Egyptian	Egyptian	Algerian (Arabic)	Algerian (Arabic)	Iranian
Age of onset	4 mo	1 mo	Infancy	2 days	Childhood	8 mo	3 days
Alive/deceased	Deceased (14 yo)	Deceased (exact age unknown; >11 yo)	Deceased (9 mo)	Deceased (18 mo)	Deceased (16 yo)	Alive (11 yo)	Deceased (5 mo)
Initial presentation	Hemolytic anemia (transfusion dependent until splenectomy) Thrombocytopenia	Hemolytic anemia (transfusion dependent until splenectomy)	Hemolytic anemia	Congenital anemia Hyperbilirubinemia Thrombocytopenia	Bronchiectasis	Neutrophilic dermatitis Fever Growth retardation Anemia	Neonatal hyperbilirubinemia Anemia
Clinical manifestations	Recurrent respiratory infections ARDS leading to death	Recurrent respiratory infections Lupus (4 yo) (arthritis, proteinuria, and hemolytic anemia)	Liver dysfunction Pneumonia	Myelodysplasia Macrocytic dyserythropoietic anemia Interstitial lung disease	Bronchiectasis ARDS Pulmonary arterial hypertension	Interstitial lung disease	Anemia Thrombocytopenia Transient neutropenia
Infectious organisms	Bacterial (*Haemophilus influenzae* sepsis, *Pseudomonas aeruginosa*) MAC pulmonary infection Aspergillus pulmonary infection Viral URIs (influenza A)	Bacterial (*Pseudomonas*) Viral URIs	No data	No data	Bacterial (pulmonary infection)	Bacterial infection	Recurrent respiratory infection (no organism isolated)
Physical exam findings	Splenomegaly Clubbing of fingers	Splenomegaly Clubbing of fingers	No data	Hepatosplenomegaly	No data	Hepatosplenomegaly Clubbing of fingers	Hypotonia Hepatosplenomegaly
Immune labs	dsDNA negative ANCA negative Coombs negative	dsDNA positive Coombs negative	No data	Coombs negative initially, then RBC autoantibody positive	No data	ANCA negative	No data
Therapies	Splenectomy for transfusion-dependent anemia (21 mo) Pulmonary treatment (7 yo) IVIG (11 yo)	Splenectomy for transfusion-dependent anemia (2 mo) IVIG (2 yo) IFNγ injection (6 yo) High-dose steroids for lupus Supplemental oxygen Pulmonary treatments	No data	Steroids	Steroids	Splenectomy (7 yo) Steroids Cyclosporine	Exchange transfusion for neonatal hyperbilirubinemia Frequent transfusion IVIG Erythropoietin GCSF

GCSF, granulocyte colony-stimulating factor; IVIG, intravenous immunoglobulin; yo, years old.

Patient A(II-1), an African American female, presented with thrombocytopenia and Coombs-negative hemolytic anemia at 4 mo and was transfusion-dependent until splenectomy. She had frequent respiratory infections in early childhood and had digital clubbing on exam. At 13 years, she was diagnosed with mycobacterial infection, followed by influenza A pneumonia complicated by bacterial superinfection, resulting in cavitary lesions and fibrosis on chest CT (Fig. 1 A). She underwent a lung lobectomy that was complicated by pleural fistulae and infections. She died at 14 years from severe ARDS, and her autopsy revealed grossly hemorrhagic lung tissue with a large aspergilloma and localized purulent pneumonia (Fig. 1, C and D). Of note, this patient developed progressive leukocytosis with associated neutrophilia in the last month of life (Fig. 1 E).

Patient B(II-3), a female of Mexican ancestry, presented at 1 mo with hemolytic anemia and remained transfusion-dependent until splenectomy. She experienced recurrent respiratory infections and had digital clubbing. Chest CT revealed areas of emphysematous lung disease (Fig. 1 A). At 4 years, she was diagnosed with lupus, with positive anti-dsDNA antibodies, arthritis, proteinuria, and Coombs-negative hemolytic anemia, and was treated intermittently with systemic steroids. She died later from infectious complications. Her older brother had hemolytic anemia since infancy, mycobacterial and fungal pneumonia, and died at 8 years.

Patient C(II-2), born to consanguineous parents of Egyptian ancestry, had a female sibling C(II-1) who died at 9 mo with severe hemolysis, liver dysfunction, and pneumonia. C(II-2) presented on day 2 of life with indirect hyperbilirubinemia, Coombs-negative anemia, required multiple transfusions, and was discharged at 6 wk. From 2 mo, he developed recurrent transfusion-dependent anemia with positive red cell autoantibodies unresponsive to steroids, thrombocytopenia, and bone marrow showing erythroid hyperplasia, megaloblastic changes, atypical megakaryocytes, and hemophagocytosis (Table S2). At 5 mo, CT chest revealed interstitial lung disease. By 11 mo, he had splenomegaly, recurrent respiratory distress, and elevated ferritin attributed to transfusions. At 15 mo, he presented with pancytopenia, hyperbilirubinemia, and elevated LDH (Table S2). Repeat bone marrow biopsy showed megaloblastic changes. He subsequently developed severe bronchospasm, pulmonary hemorrhage, and died of cardiorespiratory arrest at 18 mo.

Patient D(II-2), a male of North African descent, presented at 8 mo with anemia, neutrophilic dermatitis, finger clubbing, fever, growth retardation, and interstitial lung disease (Fig. 1 B). On skin biopsy, he had rosette-shaped bullous lesions affecting the cranial and caudal regions. Under microscopy, the epidermis showed a spongiotic appearance with associated neutrophil exocytosis. The superficial dermis was edematous and contained an intense, inflammatory, neutrophil-rich infiltrate. He underwent splenectomy and has been on long-term systemic steroids with intermittent flares. Peripheral blood analysis showed marked anemia during infancy and subsequent leukocytosis, the latter observed in the context of chronic steroid therapy (Table S2). His older sister D(II-1) had severe chronic lung disease and died from ARDS with pulmonary hypertension at 16 years.

Patient E(II-1), a male of Iranian descent, presented at 3 days of age with anemia and hyperbilirubinemia requiring exchange transfusion. He subsequently developed persistent anemia and thrombocytopenia with transient neutropenia and required intensive care unit admission for respiratory failure (Fig. 1 A and Table S2). Throughout his clinical course, he received red blood cell and platelet transfusions, as well as IVIG, erythropoietin, and G-CSF. Despite these interventions, he died at 5 mo of age from respiratory failure and presumed infection.

Together, these cases highlight a shared phenotype of infant-onset anemia and severe inflammatory lung disease.

### Immunologic abnormalities

Immunologic evaluation was available for three patients (Table 1 and Table S1). Two patients had low B cell numbers, also observed in SHP1-deficient mice (Shultz, 1988). IgG levels were normal in two patients and low in one patient, while IgA levels were elevated in two, consistent with dysgammaglobulinemia. One patient developed positive anti-dsDNA antibodies, as seen in SHP1-deficient mice (Kuntz et al., 1990). NK cell function and IFN-γ production were reduced in patient B(II-3), whose brother had died from mycobacterial and fungal infections. To gain insight into inflammatory pathways, bulk RNA sequencing (RNA-seq) was performed on peripheral blood from the patient A(II-1) and compared with a cohort of pediatric patients with septic shock and ARDS (Yang et al., 2023). Using gene set enrichment scores, this patient showed pronounced inflammatory responses, ranking in the top five for hallmark inflammatory signaling (4th out of 47), IL6/JAK/STAT3 signaling (3rd out of 47), and TNF/NF-κB signaling (3rd out of 47) (Fig. 1 F). Overall, patients had findings suggesting immune dysregulation, potentially increasing susceptibility to infection and hyperinflammatory disease.

### Genomic and in silico analyses

**Figure 3.**
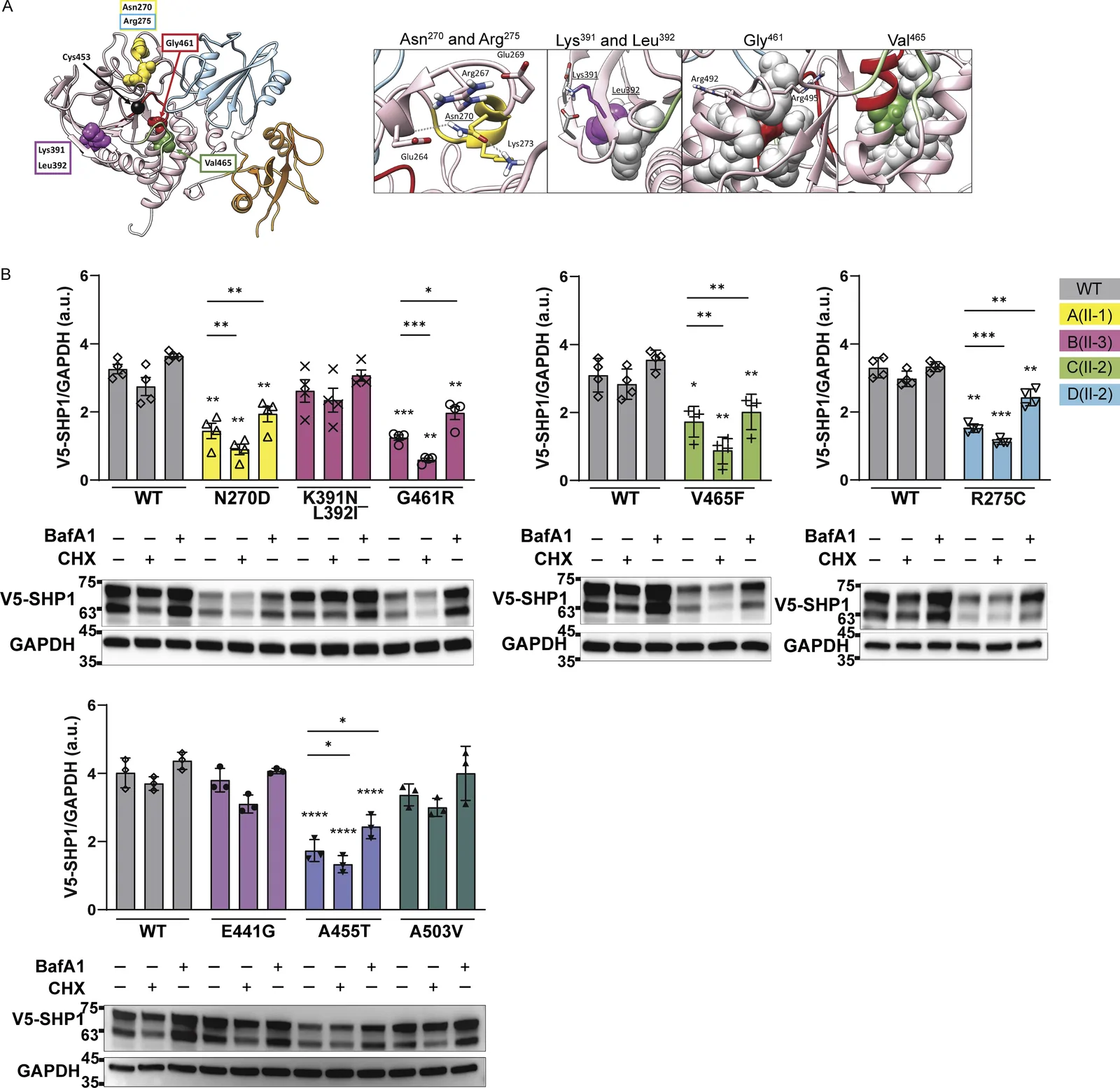
**Structural and biochemical characterization of the panel of disease-associated SHP1 mutants. (A)** Representation of the three-dimensional SHP1 structure. N-SH2, C-SH2, and PTP domains are colored light blue, orange, and pink, respectively. Residues Asn^270^ and Arg^275^ (yellow), Lys^391^ and Leu^392^ (magenta), Gly^461^ (red), Val^465^ (green), and the critical catalytic Cys^453^ (black sphere) are shown. The insets (from left to right) represent the regions surrounding Asn^270^, Lys^391^ and Leu^392^, Gly^461^, and Val^465^ in the WT protein. Residues interacting through H-bonds or electrostatically are shown in stick representation, while residues participating in hydrophobic clusters are reported in gray and in space-filling representation. H-bonds or distances between charged residues are reported as dashed gray lines. Asn^270^ makes polar interactions with Glu^264^, Arg^267^, Glu^269^, and Lys^273^. Lys^391^ forms ion pairs with Glu^384^ and Asp^386^. Leu^392^ is part of a hydrophobic core involving Tyr^390^, Trp^410^, and Tyr^412^. Gly^461^ is surrounded by hydrophobic and charged residues Arg^492^ and Arg^495^. Val^465^ is part of a hydrophobic core comprising residues Met^468^, Leu^469^, Ile^484^, Ile^488^, Val^491^, Val^499^, and Ile^508^. **(B)** Expression of V5-tagged WT and mutant SHP1 proteins in COS-1 cells at baseline and following treatment with CHX or bafilomycin (BafA1). Cell lysates were analyzed by immunoblotting, and SHP1 levels were normalized to GAPDH. The top panel includes the variants reported in this paper, while the bottom panel includes previously published variants. Asterisks indicate statistically significant differences compared with WT SHP1 under the corresponding experimental conditions, unless those referring to comparisons indicated by lines (*, P < 0.05; **, P < 0.005; ***, P < 0.001; ****, P < 0.0001; two-way ANOVA followed by Dunnett’s multiple comparison test). Source data are available for this figure: SourceData F3.

Exome sequencing of the five families revealed either compound heterozygous or homozygous variants in *PTPN6* in all seven affected individuals (Table 1 and Fig. 2, A–C). In families A–D, affected individuals carried variants that all fall within the PTP domain, whereas patient E(II-1) possessed a homozygous frameshift variant within the linker region connecting the N-SH2 and C-SH2 domains (Fig. 2 B). Specifically, patient A(II-1) carried a missense substitution (p.N270D) together with a frameshift (p.H385Afs*2) on the other allele, while patient B(II-3) had a missense variant (p.G461R) and a dinucleotide change (p.K391_L392delinsNI) in trans. In the three consanguineous families, affected individuals were homozygous for two distinct missense variants (p.V465F, family C; p.R275C, family D) and a 14-nucleotide duplication causing a frameshift variant (p.S103Tfs*3, family E). None of these variants were reported in Genome Aggregation Database (gnomAD), and all missense changes were predicted to be deleterious by CADD, REVEL, and AlphaMissense (Fig. 2 D). The affected amino acids are shown in the crystallographic structure of SHP1 in its autoinhibited state (Fig. 3 A). Both frameshift variants introduce a premature termination codon and are predicted to be LoF.

**Figure S1.**
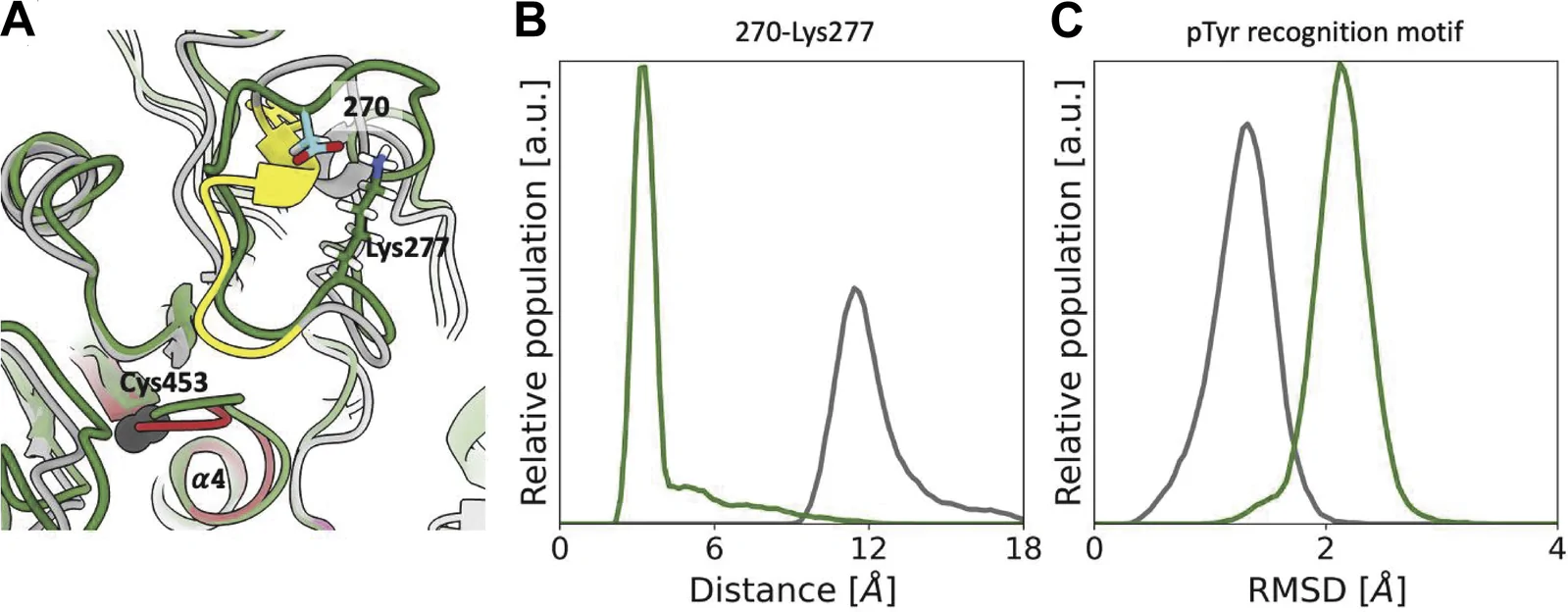
**Most representative conformation and data from the MD simulation for patient A(II-1) variant, p.Asn270Asp (N270D). (A)** Structural comparison of the region surrounding residue 270 in the most representative conformation of the WT (gray) and mutant (green) SHP1 systems. Catalytic Cys^453^ (black sphere corresponding to the α carbon), the PTP motif (red in the WT system), and the pTyr recognition motif (yellow in the WT system) are highlighted. Residues Asp^270^ and Lys^277^ are shown in stick representation and are predicted to form an ion bridge leading to a significant change in the conformation of the pTyr recognition motif, including loss of α-helical structure. **(B)** Distance between atoms C_γ_ of residue 270 (Asn or Asp) and N_ζ_ of Lys^277^, showing that Asp^270^ forms a stable ion-pair interaction with Lys^277^ (green curve), which is not present in the WT protein (gray curve). **(C)** Root mean square deviation (RMSD) from the crystallographic structure (calculated on the backbone, after fitting to the rigid protein core) of the pTyr recognition motif. The deviation of this region from the crystallographic structure was significantly larger for the simulation of the p.Asn270Asp SHP1 system (green) than for the trajectory of the WT protein (gray).

**Figure S2.**
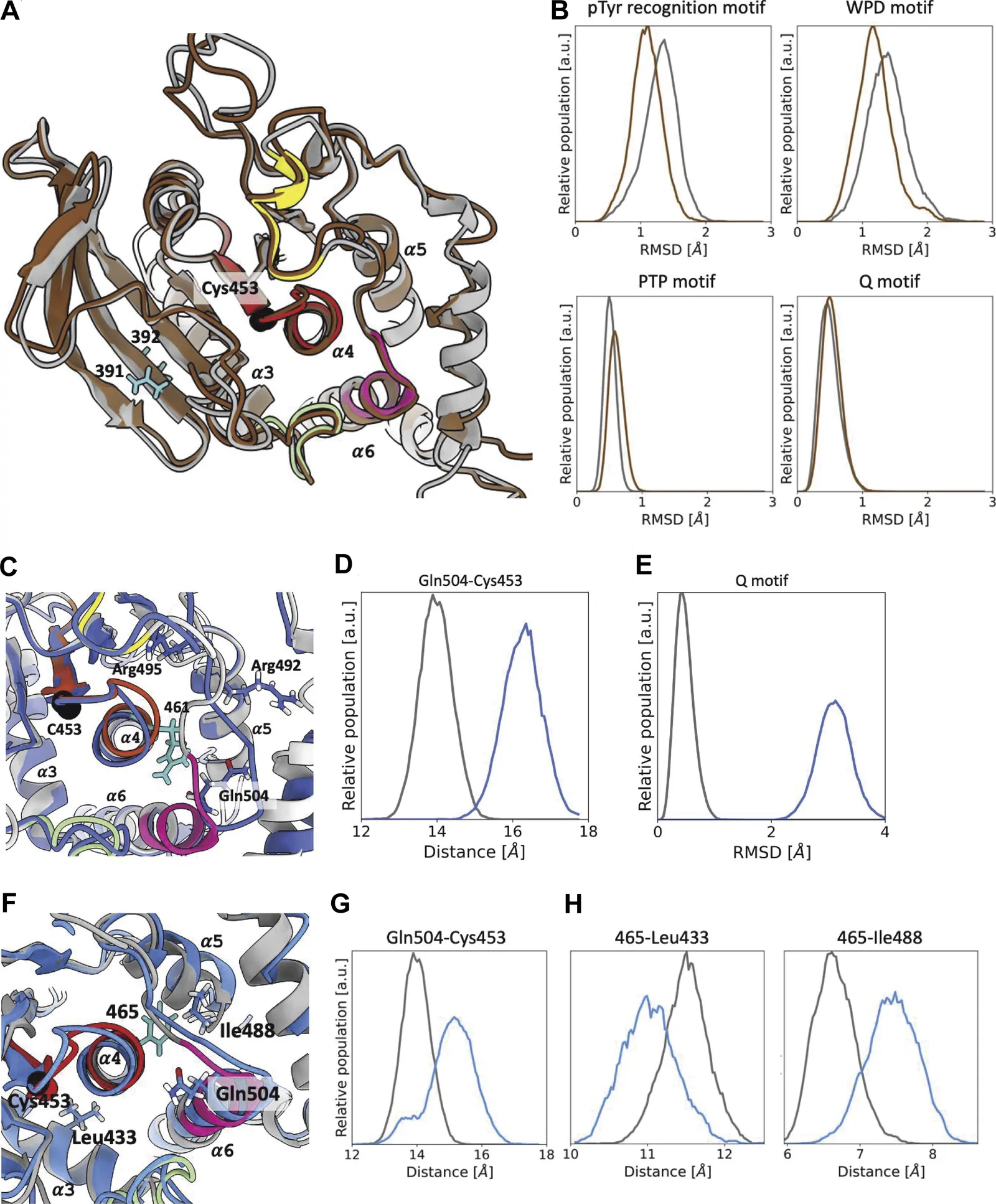
**Most representative conformation and data from the MD simulation for patient B(II-3) variant, p.Lys391_Leu392delinsAsnIle (K391N-L392I) and p.Gly461Arg (G461R), and patient C(II-2) variant, p.Val465Phe (V465F). (A)** Structural comparison of the region surrounding residues 391 and 392 and the catalytic site in the most representative conformation, along with MD simulations of the WT (gray) and mutant (brown) SHP1 systems. Catalytic Cys^453^ is indicated by a black sphere corresponding to its α carbon. PTP motif (residues 448–461), pTyr recognition motif (residues 270–276), and WPD motif (residues 417–423) are colored in red, yellow, and light green, respectively (for the WT protein). Residues Asn^391^ and Ile^392^ are shown in stick representation and colored in cyan (mutant). No significant structural variations are observed in the conserved motifs involved in catalysis. **(B)** RMSD of backbone atoms with respect to the crystallographic conformation (after fitting to the protein rigid core) of the four catalytic conserved motifs, in both the WT (gray) and mutant (brown) SHP1 proteins. No gross differences are appreciable. **(C)** Structural comparison of the region surrounding residue 461, in the most representative conformation along the MD simulations of the WT protein (gray) and Gly461Arg variant (blue). The catalytic Cys^453^ is indicated by a black sphere (corresponding to its α carbon). The PTP motif (residues 448–461) and Q motif (residues 500–507) are colored in red and pink, respectively (for the WT structure only). Residues Arg^461^ (cyan), Arg^492^, Arg^495^, and Gln^504^ are shown in stick representation (for the mutant structure only). A relative shift in the position of helices α4 and α6 (and therefore in the relative orientation of PTP and Q motifs) and a conformational transition in the Q motif are observed. **(D)** Distance between the C_α_ atoms of residues Gln^504^ and Cys^453^, critical for catalysis, for the WT (gray) and mutant (blue) SHP1 simulations. **(E)** RMSD of Q motif backbone atoms with respect to the crystallographic conformation (after fitting to the protein rigid core) of the four catalytic conserved motifs, in both the WT (gray) and mutant (blue) SHP1 simulations. **(F)** Structural comparison of the region surrounding residue 465, in the most representative conformation along the MD simulations of the WT protein (gray) and Val465Phe variant (light blue). The catalytic Cys^453^ is indicated by a black sphere (corresponding to its α carbon). The PTP motif and Q motif are colored red and pink, respectively (for the WT structure only). Residues Phe^465^ (cyan), Leu^433^, and Ile^488^ are shown in stick representation (for the mutant structure only). A relative shift in the position of helices α4 and α6 (and therefore in the relative orientation of PTP and Q motifs) is observed. **(G)** Distance between the C_α_ atoms of residues Gln^504^ and Cys^453^, critical for catalysis, for the WT (gray) and mutant (light blue) SHP1 simulations. **(H)** Distance between the C_α_ atoms of residue 465, in helix α4, with Leu^433^, in helix α3 (left), and Ile^488^, in helix α5 (right), for the WT (gray) and mutant (light blue) SHP1 simulations. RMSD, root mean square deviation.

To assess the variants’ impact on protein function, molecular dynamics (MD) simulations were performed on the isolated PTP domain of SHP1 (Fig. S1). Residues Asn^270^ and Arg^275^ are part of a conserved motif for binding phosphotyrosine substrates (pTyr) (Yang et al., 2003). Specifically, Arg^275^ is conserved in all human PTP proteins, and substitutions at Arg^275^ are predicted to abolish catalytic function (Andersen et al., 2001). In MD simulations, the Asn-to-Asp substitution (p.N270D) caused a marked conformational change in this region (Fig. S1). Substitutions at residues Lys^391^ and Leu^392^ slightly perturbed a region outside the catalytic site (Fig. S2) but offered no insight into their effect on protein function. Residues Gly^461^ and Val^465^ lie within or adjacent to the conserved PTP signature motif, which contains the catalytic Cys^453^ residue. MD simulations predicted that both amino acid substitutions (p.G461R and p.V465F) would lead to major structural perturbations near the catalytic active site (Fig. S2). Overall, the *PTPN6* variants are rare and predicted to impair the function of SHP1, with the possible exception of p.K391_L392delinsNI.

### Biochemical and functional analyses

To validate the disruptive consequences of the variants, stability and catalytic activity of each SHP1 mutant were assessed relative to WT. Each SHP1 variant was transiently expressed in COS-1 cells, and protein levels were measured at baseline and following treatment with the protein synthesis inhibitor cycloheximide (CHX) (Fig. 3 B). Immunoblotting and relative quantitative analyses revealed a significant reduction in SHP1 protein levels for mutants SHP1^N270D^, SHP1^G461R^, SHP1^V465F^, and SHP1^R275C^. In line with the structural predictions, SHP1^K391_L392delinsNI^ (K391N_L392I) had only a minor effect on protein stability. Bafilomycin A1 (BafA1) treatment rescued mutant protein levels consistent with autophagy/lysosomal degradation, likely due to aberrant protein folding. Protein stability was also investigated for two missense variants previously associated with neutrophilic dermatosis (p.E441G) and smoking-associated early-onset emphysema (p.A455T), as well as a rare homozygous missense variant found in gnomAD (p.A503V). SHP1^A503V^ showed similar stability to the WT protein. The variant p.E441G displayed levels comparable with SHP1^WT^ and SHP1^A503V^, while p.A455T resulted in significantly reduced protein levels in line with previous observations (Bossé et al., 2019).

**Figure 4.**
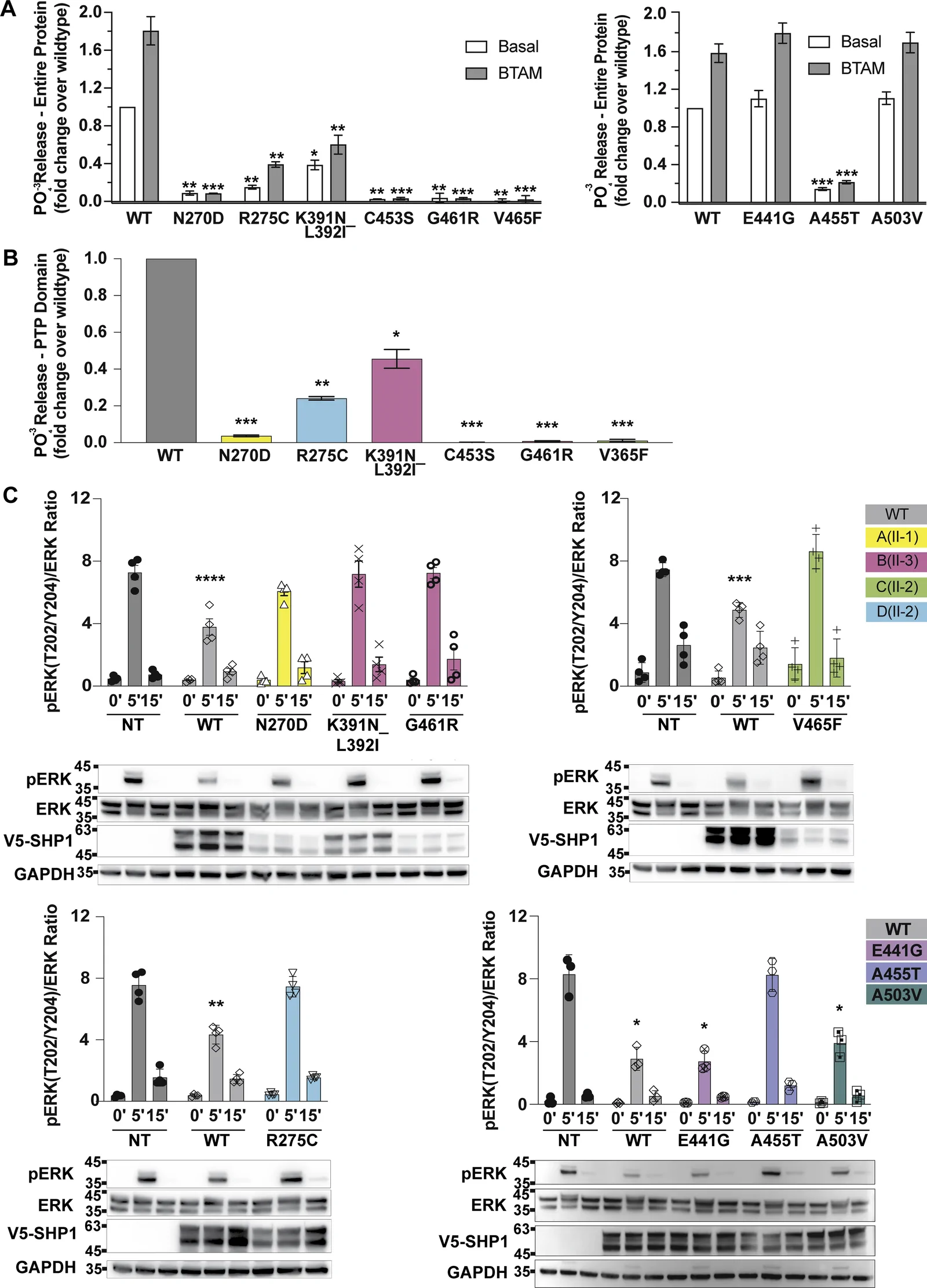
**Phosphatase activity and MAPK signaling modulation of disease-associated SHP1 mutants. (A)** Full-length SHP1 phosphatase activity was assessed at baseline and following stimulation with the activating BTAM peptide. **(B)** In vitro phosphatase activity of WT and mutant SHP1 catalytically active form lacking the autoinhibitory N-SH2 domain. **(C)** HEK293T cells overexpressing WT or mutant variants were treated with EGF (5 and 15 min), and lysates were analyzed for ERK phosphorylation. Representative immunoblots and mean ± SEM densitometry values are shown. Asterisks indicate statistically significant differences in ERK phosphorylation levels compared with control cells (NT, mock transfection) at the corresponding experimental points (*, P < 0.05; **, P < 0.005; ***, P < 0.001; ****, P < 0.0001; two-way ANOVA followed by Dunnett’s multiple comparison test). Source data are available for this figure: SourceData F4.

To assess the catalytic activities of the individual protein variants, full-length WT and mutant *PTPN6* constructs (Fig. 4 A) and constructs lacking the region encoding the autoinhibitory N-SH2 domain (Fig. 4 B) were expressed in bacteria, and the catalytic activity of each protein was measured in vitro at baseline and after stimulation with a bisphosphoryl tyrosine–based activation motif (BTAM) activating peptide. SHP1^N270D^ showed a markedly reduced catalytic activity, whereas the SHP1^G461R^ and SHP1^V465F^ mutants were catalytically inactive. Both SHP1^R275C^ and SHP1^K391_L392delinsNI^ exhibited a milder but statistically reduced phosphatase activity. A significantly reduced catalytic activity was also documented for the smoking-associated early-onset emphysema SHP1^A455T^ mutant, while SHP1^E441G^ and SHP1^A503V^ displayed similar phosphatase activity to WT SHP1.

**Figure S3.**
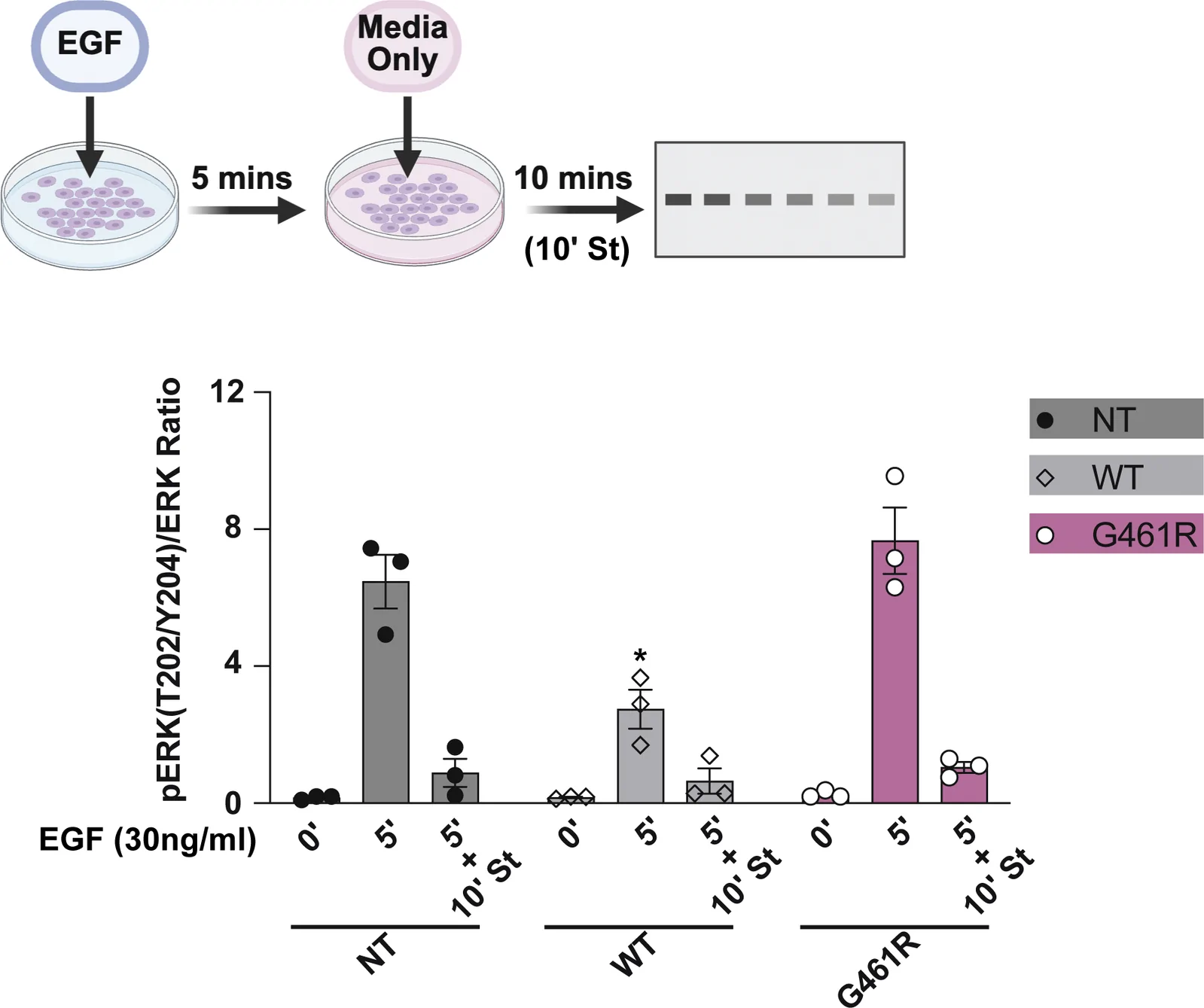
**Time-course ERK phosphorylation assay.** HEK293T cells were transfected to express SHP1^G461R^. After 24 h, cells were starved (16 h) and then stimulated with EGF (30 ng/ml), which was removed after 5 min, as indicated in the cartoon. The graph shows the levels of activated ERK (pERK) at different time points. A LoF behavior of the mutant is observed (pERK dynamics similar to that observed in non-transfected cells [NT]). The asterisk indicates the statistically significant difference in ERK phosphorylation levels compared with control cells (NT, mock transfection) at the corresponding experimental point. (*, P < 0.05, two-way ANOVA followed by Tukey’s comparison test). Schematic created with https://BioRender.com/j8nvxcj. Source data are available for this figure: SourceData FS3.

Finally, to further validate the LoF behavior of the identified missense variants, the inhibitory role of SHP1 on MAPK signaling was investigated. Transfected HEK293T cells were stimulated with epidermal growth factor (EGF) in a time-course experiment. In contrast to WT and the variants p.E441G and p.A503V, all of the variants linked with an ARDS phenotype, including the p.A455T variant, failed to downmodulate pERK signaling (Fig. 4 C). To assess signal termination, we additionally measured pERK decay following EGF withdrawal for non-transfected control, SHP1^WT^, and SHP1^G461R^. Unlike WT, SHP1^G461R^ was unable to downmodulate ERK, consistent with LoF (Fig. S3).

### Concluding remarks

We describe the first cohort of patients harboring biallelic coding variants in *PTPN6* that result in loss of SHP1 function, establishing a previously unrecognized monogenic disorder characterized by infant-onset anemia and severe inflammatory lung disease. Previous evidence linking *PTPN6* to human disease has been limited to two reports. One individual carrying compound heterozygous variants consisting of a missense change (p.E441G) and an intronic deletion within the promoter region of *PTPN6* was reported with Sweet’s syndrome, a form of neutrophilic dermatosis. cDNA analysis suggested aberrant *PTPN6* transcript processing (Nesterovitch et al., 2011). Our functional studies on the p.E441G variant did not demonstrate impaired SHP1 stability, catalytic activity, or ERK regulation, suggesting that the pathogenic effect, if any, may largely derive from the accompanying promoter-region deletion. The second report identified a heterozygous p.A455T variant in one family with incomplete penetrance of early-onset emphysema (Bossé et al., 2019). Consistent with the original report, we found that p.A455T reduced SHP1 stability and catalytic activity and failed to suppress ERK signaling. This variant therefore likely causes a distinct, dominantly inherited form of SHP1 deficiency with incomplete penetrance, though whether it arises through haploinsufficiency or a dominant-negative effect remains to be determined. Together with the seven patients described here, these findings establish SHP1 deficiency as a cause of severe inflammatory and hematologic disease in humans.

The variants in our cases constitute an allelic series ranging from catalytically inactive to hypomorphic, yet even the hypomorphic alleles were associated with severe disease. The p.R275C variant had reduced catalytic activity with reduced protein stability and failed to downmodulate ERK signaling. The p.K391_L392delinsNI variant had normal protein stability and a modest reduction in catalytic activity but an inability to dampen ERK signaling. The combination of this variant in trans with the catalytically inactive p.G461R variant likely produced little functional SHP1 activity resulting in severe disease. Together, these findings demonstrate that severe clinical disease can result across a continuum of SHP1 dysfunction, ranging from complete catalytic inactivation to hypomorphic alleles.

Among the clinical features in our patients, ARDS and chronic lung disease were among the most life-threatening. The predominance of neutrophilic infiltrates in the lung, together with prior murine studies demonstrating inflammatory lung disease in SHP1-deficient mice lacking B and T cells (Yu et al., 1996), supports innate inflammation as a likely primary driver of pulmonary pathology, although adaptive immune mechanisms may also contribute. To date, *PTPN6* variants have not been implicated in ARDS cohorts, though studies largely involve individuals of European ancestry (Cao et al., 2024), whereas our cohort spans diverse ancestral backgrounds. It is also possible that *PTPN6* variants contribute more broadly to inflammatory lung disease, including ARDS, as suggested by the p.A455T variant in the family with early-onset emphysema (Bossé et al., 2019). Furthermore, given the lung involvement in our cohort plus the preclinical model showing SHP1 agonists mitigating neutrophil-driven lung injury (Moussavi-Harami et al., 2024), SHP1 activation may be worth investigating as a therapeutic strategy for SHP1-related inflammatory lung disease and, potentially, the broader spectrum of neutrophil-driven inflammatory lung diseases, including ARDS.

The other severe phenotype in our cohort was Coombs-negative hemolytic anemia during infancy, with most requiring splenectomy and ongoing periodic transfusions. This is likely due to the role of SHP1 in CD47-SIRPα signaling, where CD47 expressed on erythrocytes acts as a “do not eat me” signal through its interaction with the SIRPα receptor on resident macrophages, recruiting SHP1, which transmits an inhibitory signal that suppresses RBC phagocytosis (Barclay and Brown, 2006). Thus, in SHP1 deficiency, the impaired inhibitory signaling through the CD47-SIRPα axis can result in antibody-independent hemolysis. Furthermore, the hemolytic anemia in motheaten mice has been reported to be secondary to increased oxidative stress, leading to the oxidation of phospholipids on the erythrocyte membrane, causing hemolysis (Lyons et al., 2003). Together, impaired CD47-SIRPα signaling and heightened oxidative stress likely drive the predominantly antibody-independent hemolysis in our patients, although the later development of autoantibodies in one patient suggests autoimmune mechanisms may also contribute in some cases.

Collectively, these findings establish impaired SHP1-mediated inhibitory signaling as a unifying mechanism underlying the pulmonary and hematologic manifestations of this newly recognized disorder. As SHP1-directed therapeutics emerge for cancer and inflammatory disorders, these observations provide an important human genetic framework for understanding the biological consequences of modulating SHP1 signaling and for guiding the rational development and application of SHP1-targeted therapeutic strategies.

## Materials and methods

### Study oversight

Written informed consent was obtained from all individuals (parents and parents on behalf of patients from each family) for genetic testing and the use of clinical information and images in this study, according to the ethical approval of the local Institutional Review Boards (IRBs) at University of California, San Francisco (18-25891), National Institutes of Health (01-I-0202), CHU Montpellier (DC-2024-6611), and King Abdullah University of Science and Technology (23IBEC090).

### Next-generation sequencing and analysis

#### Family A (c.808A>G, p.N270D; c.1151dupA, p.H385Afs*2)

Exome sequencing was obtained in a CLIA-approved lab. Exon regions were targeted in extracted genomic DNA from patient and family members using the xGen Whole Exome Panel kit (Integrated DNA Technologies). Targeted regions were sequenced using the Illumina HiSeq 2500 sequencing system (v3 chemistry) with 100-bp paired-end reads in rapid run mode. The resulting DNA sequences were mapped to and analyzed in comparison with the published human genome reference genome GRGh37/hg19. Sequence variants were filtered, ranked, and annotated using Opal Clinical (Fabric Genomics) and Moon Diploid and compared with other provided family members to search for causes of Mendelian genetic disease (de novo, homozygous, compound heterozygous, and inherited heterozygous disease-causing variants). All pathogenic, likely pathogenic, and clinically suspicious variants of uncertain significance are reported. All reported variants are confirmed by Sanger sequencing.

#### Family B (c.1173_1174delinsTA, p.K391_L392delinsNI; c.1381G>C, p.G461R)

Sequencing libraries were generated using Illumina’s TruSeq DNA sample preparation kit and the recommended protocol for the gel-free method for TruSeq Enrichment. Generated TruSeq libraries were assessed using the 2100 Bioanalyzer and High Sensitivity DNA Assay chip (Agilent Technologies). Exome-enriched libraries were sequenced on Illumina HiSeq 2000. Identified sequences were mapped to the human genome reference consortium GRGh37/hg19 using Bowtie 2 (Langmead et al., 2009). Multiply mapped reads and PCR duplicates were removed using SAMtools (Sequence Alignment/Map) (Li et al., 2009). Additional filtering of the pre-aligned BAM files to remove reads with low-quality mapping and alignment scores was performed using Avadis NGS version 1.4.6 software (Strand Scientific Intelligence, Inc.). Identification of SNPs and indels was performed using Avadis NGS software.

#### Family C (c.13893G>T, p.V465F, homozygous)

Double-stranded DNA capture baits against ∼36.5 Mb of the human coding exome (targeting >98% of the coding RefSeq from the human genome build GRCh37/hg19) were used to enrich target regions from fragmented genomic DNA with the Twist Human Core Exome Plus kit. The generated library was sequenced on an Illumina platform to obtain at least 20× coverage depth for >98% of the targeted bases. An in-house bioinformatics pipeline, including read alignment to the GRCh37/hg19 genome assembly, variant calling, annotation, and comprehensive variant filtering, was applied. All variants with a minor allele frequency (MAF) of <1% in the gnomAD database, and disease-causing variants reported in the Human Gene Mutation Database (HGMD), ClinVar, or CENTOGENE’s Biodata Bank were considered. The coding exons and flanking ±20 intronic nucleotides of genes with documented gene-phenotype evidence (based on, but not limited to, OMIM information).

#### Family D (c.823C>T p.R275C, homozygous)

Trio exome sequencing was performed on blood-derived DNA samples using the Twist Human Core Exome EF Multiplex Complete Kit (Twist Bioscience) according to the manufacturer’s instructions. 75-bp paired-end reads were generated on an Illumina NextSeq500 (Illumina). FASTQ files were aligned to the human genome GRCh37/hg19 with Burrows-Wheeler Aligner (version 0.7.15). Variants were annotated using ANNOVAR and filtered using in-house scripts to keep variants impacting exonic sequences or splice sites ( ±10 bp from the junction) and with an allele frequency <1% in the 1000 Genomes Project, gnomAD version 2, and in a local database. Priority was given to rare exonic or donor/acceptor splicing variants in accordance with the pedigree and phenotype, fitting a recessive (homozygous or compound heterozygous) model or variants in genes previously linked to inflammatory disorders or dermatological conditions.

#### Family E (c.292_305dupTACCCGCTGAACTG, p.S103Tfs*3, homozygous)

Exome sequencing was accomplished on genomic DNA extracted from the peripheral blood of the patient. Following library preparation with SureSelect V7-Post, sequencing was conducted on a NovaSeq6000 platform producing 101-bp paired-end reads with a minimum coverage of 100× for 85% of reads. The resulting reads were aligned to the GRCh37/hg19 reference genome, and variants were called and filtered using standard bioinformatics tools (BWA, GATK). Variants were annotated with ANNOVAR and prioritized based on clinical relevance and allele state using ClinVar. Following the autosomal recessive inheritance model, allele read frequencies of 75–100% were considered for homozygous alterations, while allele frequencies below 1% were applied for recessive variants. Variant assessment was performed using the HGMD and the dbSNP repository. To determine MAF (MAF < 0.01), data from the gnomAD version 2.1.1 (https://gnomad.broadinstitute.org/) were referenced. Following exome data analysis, gene variants were systematically filtered and cross-checked against multiple databases, including dbSNP (https://www.ncbi.nlm.nih.gov/snp/), the 1000 Genomes Project (https://www.internationalgenome.org/), and gnomAD (https://gnomad.broadinstitute.org/).

### Histology and immunofluorescence staining

Lung tissue was fixed in 10% formalin and embedded in paraffin. H&E stained sections were imaged as part of the autopsy. For immunofluorescence, paraffin-embedded blocks were cut into 4-µm thick sections, dewaxed in xylene, rehydrated in ethanol, and antigen retrieval was performed by boiling for 30 min in Diva Decloaker (Biocare Medical). Sections were blocked with 3% donkey serum, incubated overnight at 4°C with primary antibody targeting myeloperoxidase (PA5-16672; Invitrogen, Lot ZB4215629) at 1:100 with 1% donkey serum, and 0.1% Triton X-100 in phosphate-buffered saline (PBS). After washing, samples were incubated with Cy3-conjugated crossed-adsorbed polyclonal secondary antibodies targeting rabbit IgG (711-165-152; Jackson Immuno Research, Lot161128) at 1:250 for 1 h at room temperature. After additional washes, sections were stained with DAPI nuclear stain at 1:1,000 (D1306; Invitrogen, Lot 2942291, 5 mg/ml stock concentration) for 5 min at room temperature. After additional washes, sections were mounted in Vectashield (cat no. H-1700; Vector Laboratories) for standard fluorescent imaging.

### Structural analysis and MD simulations

The starting structure for the simulation of the WT PTP domain (PTP-SHP1) was taken from the RCSB Protein Data Bank (PDB code 3PS5). The original crystallographic structure showed no gaps in the resolved residues but presented a serine in place of the catalytic cysteine. To substitute Ser453Cys, the identified in-frame indel (SHP1^K391_L392delinsNI^) and missense variants (SHP1^N270D^, SHP1^G461R^, and SHP1^V465F^), the side-chain conformation for substituted residues was chosen by selecting the most probable rotamer from the backbone-dependent rotamer library in the UCSF Chimera software package (Pettersen et al., 2004). MD simulations were performed using the GROMACS simulation package (Abraham et al., 2015). All the simulated systems (about 70,000 atoms) were first equilibrated following previously reported protocols (Martinelli et al., 2020). The PME algorithm was used for electrostatic interactions with a 1.5 nm cutoff. A reciprocal grid of 80 × 80 × 80 cells was used with 4th-order B-spline interpolation. A 1.57 nm cutoff was used for Van der Waals interactions. Starting from the equilibrated configuration, each system was studied by a single production run of 200 ns. Structural conformations of WT and mutant SHP1 systems were compared through the calculation of backbone RMSD in specific regions. The calculation of RMSDs was performed after having removed all the protein global roto-translational motions by fitting to the initial conformation of the protein rigid-core (residues 226–231, 242–250, 261–267, 270–276, 280–289, 300–311, 319–352, 355, 360–365, 371–374, 377–385, 389–399, 406–439, 447–475, and 483–520). The most representative conformation of each MD simulation was obtained by clustering structures with the linkage algorithm and with a backbone RMSD cutoff of 0.5 nm.

### Biochemical analyses

The entire *PTPN6* coding sequence was cloned into a pcDNA6/V5-HisA eukaryotic expression vector (Invitrogen). Mutations were introduced by site-directed mutagenesis, using the QuikChange II Site-Directed Mutagenesis kit (Stratagene). Following polyethylenimine (Polysciences) transfection, COS-1 cells were maintained in basal conditions or treated with CHX (10 µg/ml; 2 h) or BafA1 (200 nM; 5 h) (Sigma-Aldrich) to assess protein stability and degradation pathways by western blotting using commercially available antibodies (see below). WT SHP1 and each mutant were also transiently overexpressed in HEK293T cells to assess their effects on ERK1/2 phosphorylation, following overnight starvation and EGF (Thermo Fisher Scientific) stimulation (30 ng/ml, 5 and 15 min), using commercially available antibodies (see below). Full-length SHP1 proteins and their catalytically active domain lacking the autoinhibitory N-SH2 domain (C-SH2-PTP-SHP1) were expressed in bacteria (pET-26b vector, Novagen) and purified, as previously reported (Martinelli et al., 2008). Catalytic activities were measured in vitro, basally (C-SH2-PPTP-SHP1 and whole protein) and following stimulation with a BTAM (whole protein). Primary antibodies used included: mouse monoclonal anti-V5 (Invitrogen), mouse monoclonal anti-GAPDH (Santa Cruz Biotechnology), rabbit polyclonal anti-p44/42 MAPK1/3 (ERK 1/2), and mouse monoclonal anti-phospho-p44/42 MAPK1/3 (ERK 1/2) (Thr202/Tyr204) (Cell Signaling Technology).

### Bulk RNA-seq of pediatric cohort with sepsis and ARDS

Cohort 1 included 46 children with septic shock enrolled in a larger trial of tight glycemic control in critically ill children (NCT01565941). As previously reported (Yang et al., 2023), all patients were mechanically ventilated, had a documented clinical diagnosis of sepsis, and were receiving vasoactive infusions for no more than 3 days. Whole blood was collected in PAXgene tubes on the day of two blood glucose levels >150 mg/dl, which was within the first 3 days of sepsis onset. cDNA libraries were prepared using the Nugen Universal Plus kit with PolyA capture (Nugen), and 150-nucleotide paired-end libraries were sequenced on a NovaSeq S4 system (Illumina) to a target depth of 40–50 million read pairs per sample. Gene counts were produced using HTSeq version 0.13.5.

Cohort 2 included *n* = 71 immunocompromised children with respiratory disease enrolled in a prospective observational study at UCSF (IRB 14-13546). All patients were mechanically ventilated for pulmonary disease. Whole blood was collected in PAXgene tubes on the day of clinically indicated bronchoscopy. Samples were processed at UCSF, where RNA was extracted using the Quick-RNA Miniprep Plus Kit (Zymo) with DNase, Proteinase K, and FastSelect treatment. cDNA libraries were prepared with the NEBNext Ultra II RNA Library Prep Kit (New England Biolabs), and 150-nucleotide paired-end libraries were sequenced on a NovaSeq S6 system (Illumina) to a target depth of 40 million read pairs per sample. Gene counts were produced using STAR. Next, count matrices from both cohorts were merged to a core of 14,528 detected genes, normalized using CombatSeq to account for batch-specific differences, and further normalized using a variance-stabilizing transformation (DESeq2). Gene set enrichment scores to the MSigDB Repository were created (GSVA) and compared for septic shock patients in cohort 1 and the patient of interest (AII-1) in cohort 2.

### Online supplemental material

Figs. S1 and S2 include all the MD analyses for the variants. Fig. S3 shows time-course ERK phosphorylation assay. Table S1 shows additional laboratory studies. Table S2 shows peripheral blood counts of the patients.

## Supplementary Material

Table S1 shows additional laboratory studies.

Table S2 shows peripheral blood cell counts for patients C(II-2), D(II-2), and E(II-1), indicating anemia during infancy.

SourceData F3 is the source file for Fig. 3.

SourceData F4 is the source file for Fig. 4.

SourceData FS3 is the source file for Fig. S3.

## Data Availability

All data are available in the published article and its online supplemental material. RNA-seq data are available through a controlled access dbGaP repository (https://dbgap.ncbi.nlm.nih.gov/beta/study/phs003016.v1.p1/#study). Additional data are available from the corresponding author upon reasonable request.
