# Supplementary material for: Biallelic loss-of-function PTPN6 variants lead to inflammatory lung disease and hemolytic anemia
Source: J Exp Med. 2026 Oct 7;223(11):e20252379. doi: 10.1084/jem.20252379 (PMC13644687; doi:10.1084/jem.20252379)
Supplement: Supplementary file 2 [file jem_20252379_sourcedataf4.pdf]

F4, C (left panel)

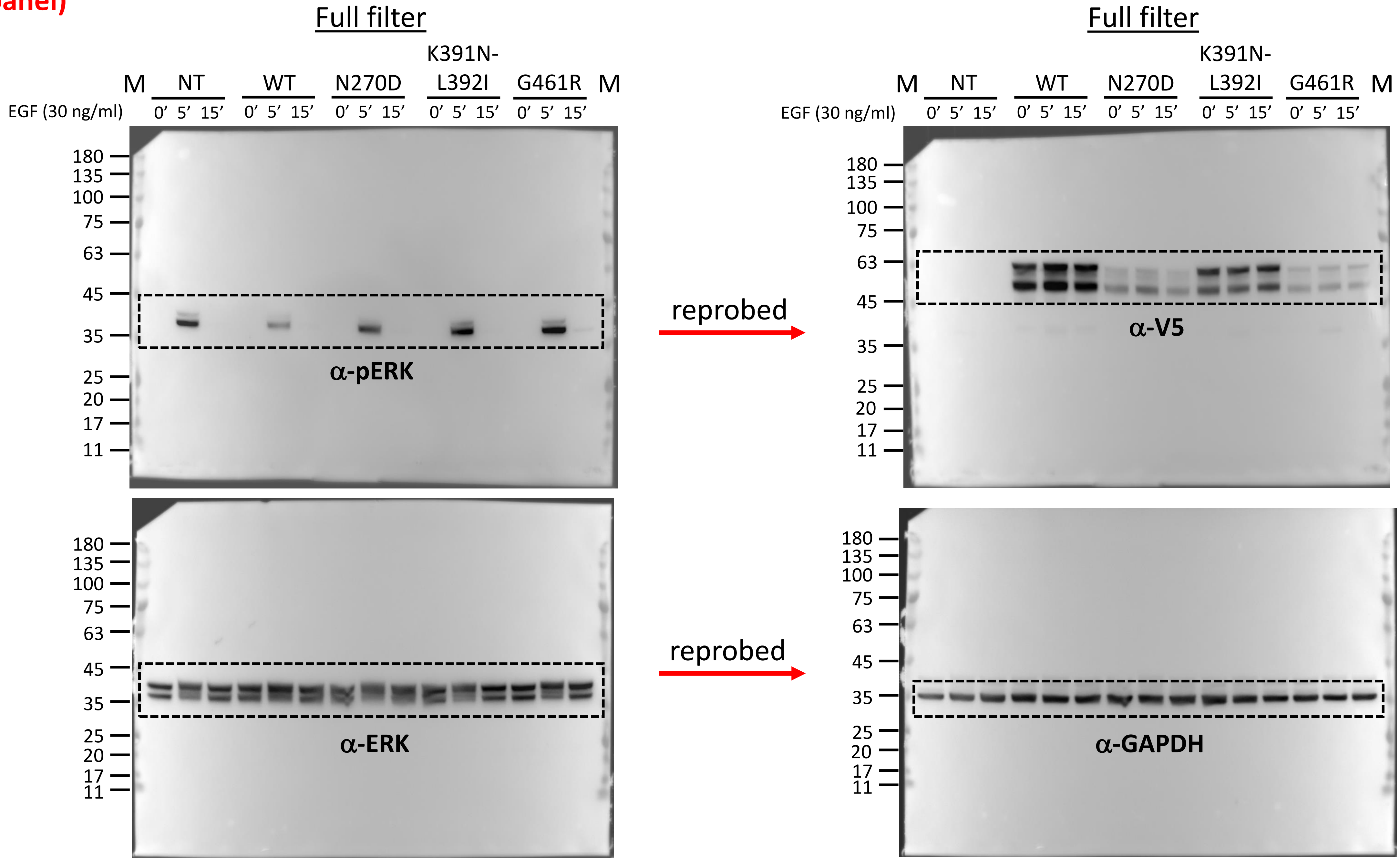

M=Markers in kDa

F4, C (right panel)

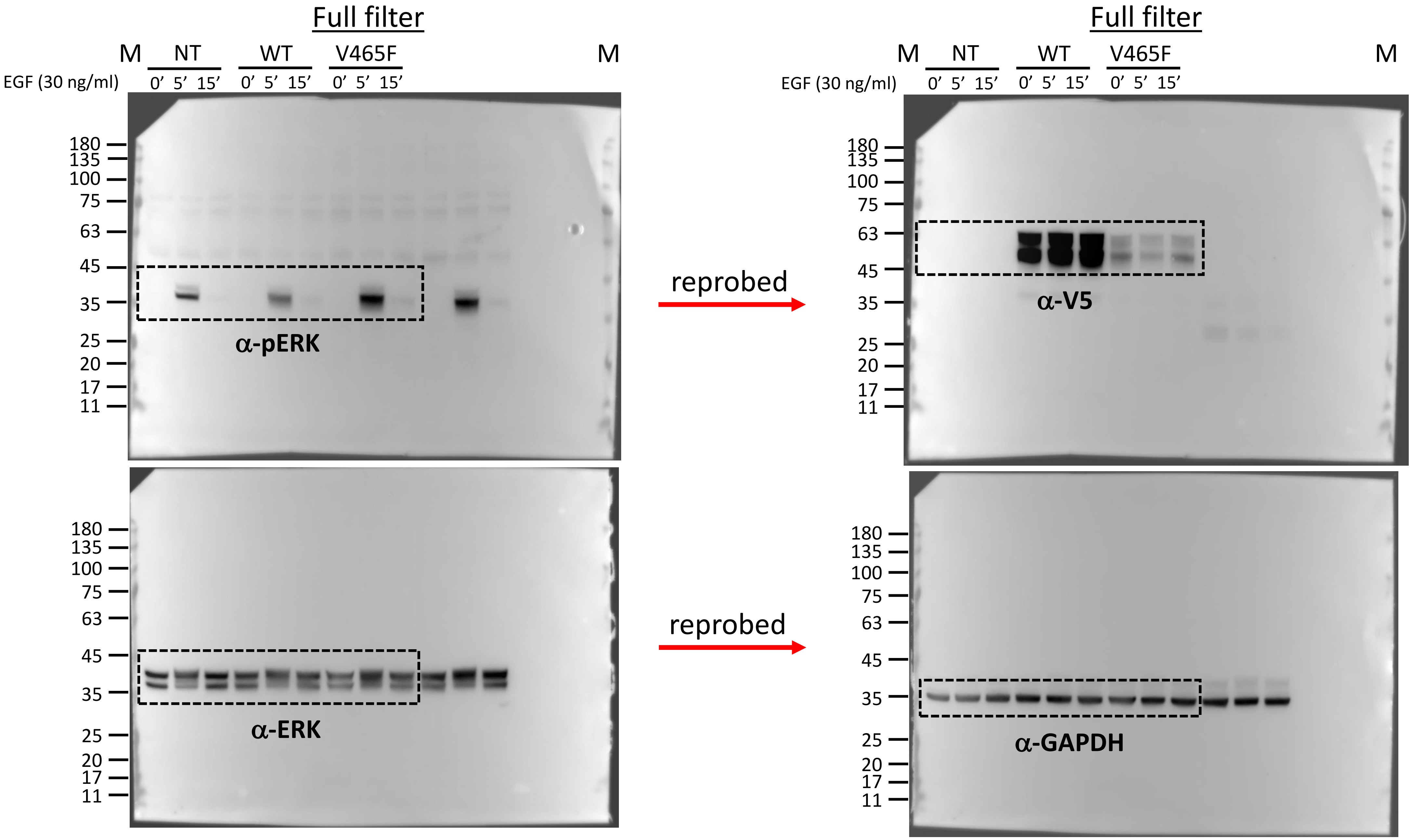

M=Markers in kDa

F4, C (left lower panel)

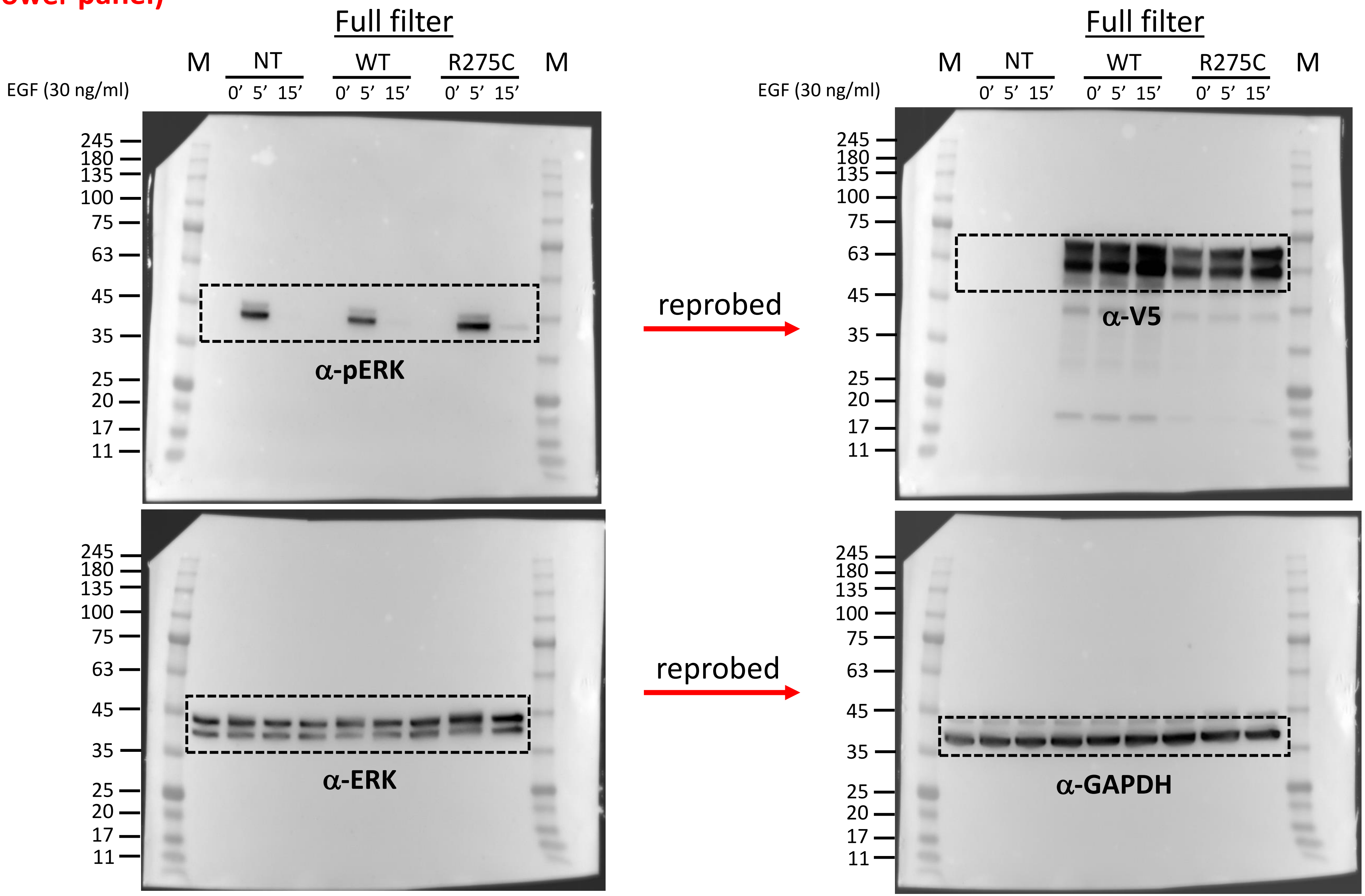

M=Markers in kDa

F4, C (right lower panel)

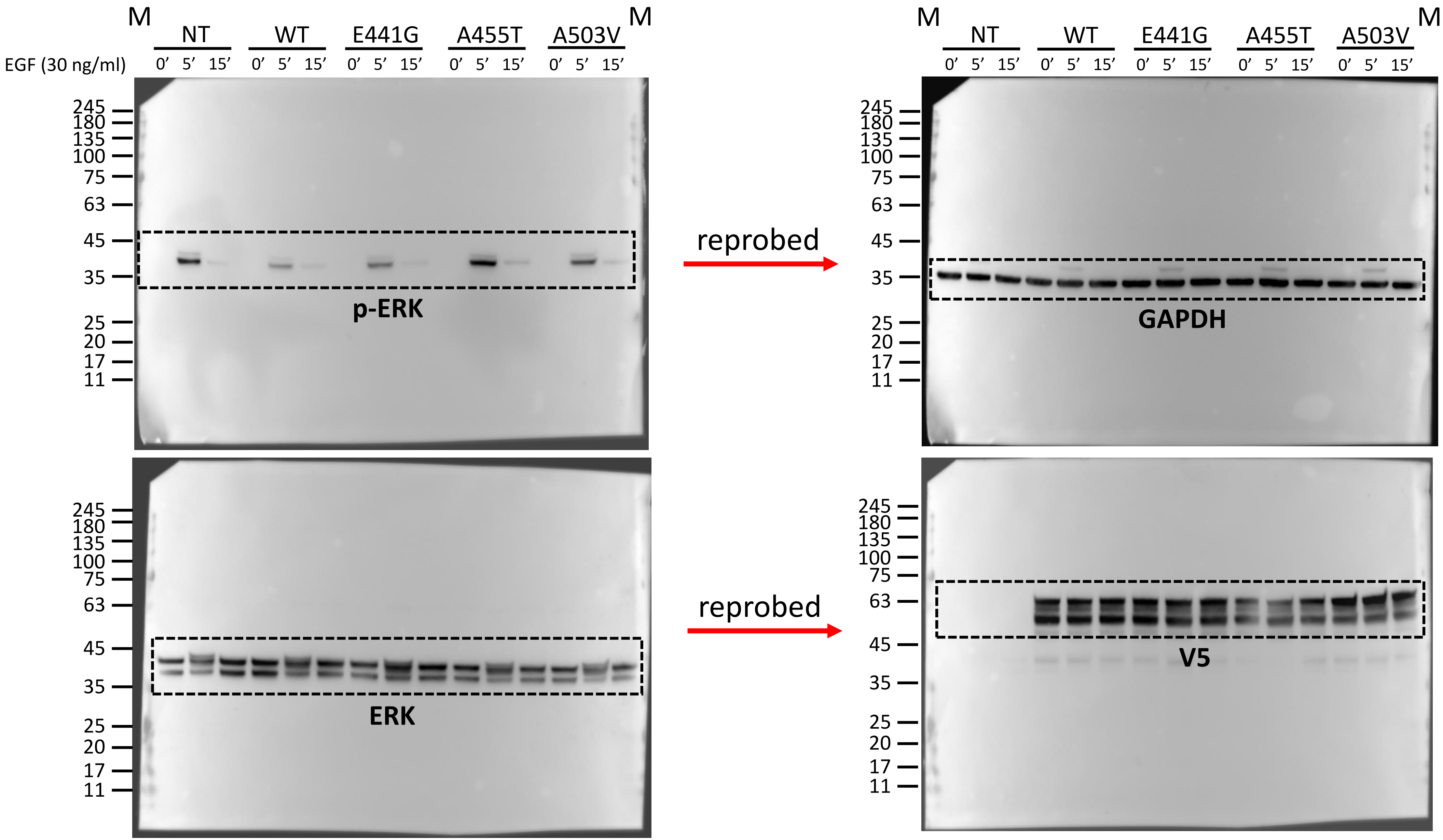

M=Markers in kDa
