# Supplementary material for: Biallelic loss-of-function PTPN6 variants lead to inflammatory lung disease and hemolytic anemia
Source: J Exp Med. 2026 Oct 7;223(11):e20252379. doi: 10.1084/jem.20252379 (PMC13644687; doi:10.1084/jem.20252379)
Supplement: Supplementary file 3 [file jem_20252379_sourcedatafs3.pdf]

Supplementary Figure 3 - time-course experiments

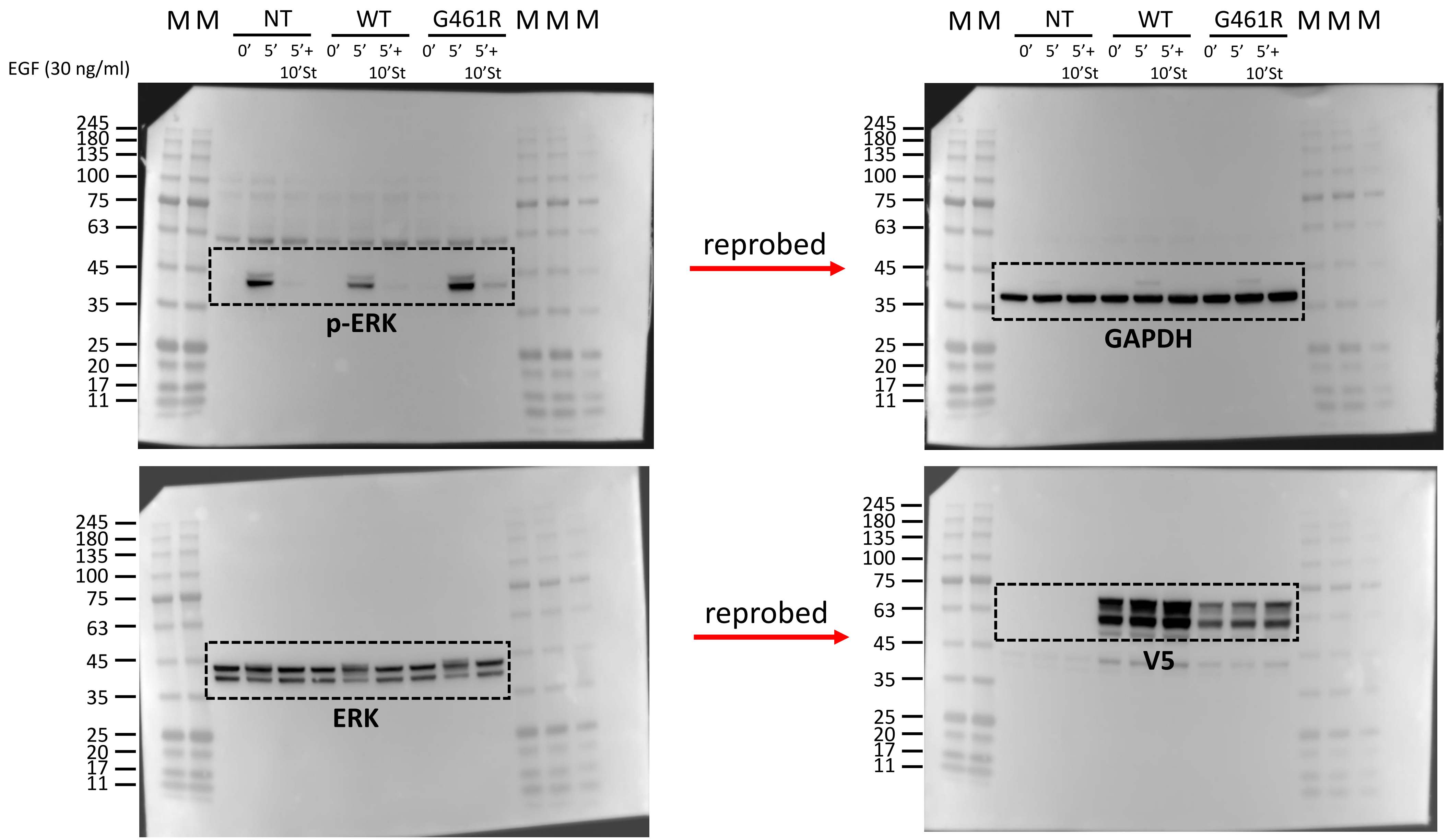

M=Markers in kDa
