# Supplementary material for: Biallelic loss-of-function PTPN6 variants lead to inflammatory lung disease and hemolytic anemia
Source: J Exp Med. 2026 Oct 7;223(11):e20252379. doi: 10.1084/jem.20252379 (PMC13644687; doi:10.1084/jem.20252379)
Supplement: Supplementary file 4 [file jem_20252379_tables1.docx]

Table S1. Additional laboratory studies.

| **A (II-1)** | **B (II-3)** | **C (II-2)** | **E (II-1)** |
| --- | --- | --- | --- |
| IgM 26 low for age  IgG low*  IgA 663 elevated for age  Lymphocyte subsets –CD3 1149 (76%), CD4 697 (47%), CD8 361 (24%), CD19 99 (6%), CD16/56 238 (15%) normal except low B cells for age  Mitogen proliferation low to PHA, ConA, PWM**  Antigen proliferation absent to tetanus**  Tetanus titer normal  Pneumococcal titers normal  CXCL9 277 pg/mL (<=121 178)^#^  IL18 2695 pg/mL (89-540)^#^  ANCA negative  SS-A, SS-B, RNP, smith, chromatin, ribosomal, centromere, Sm/RNP, Scl-70, Jo-1 antibodies negative  IFNg autoantibody negative  GMCSF autoantibody negative | IgM 74 normal for age  IgG elevated*  IgA 740 elevated for age  NK cell function decreased*  IFNg production decreased*  Lymphocyte subsets – CD3 increased, CD20 B cells low, CD16/56 normal for age*  Mitogen proliferation low to PHA; normal to PWM/ConA  Antigen proliferation normal to tetanus  CH50, C3, C4 all decreased | IgM, IgG normal for age  Normal T, B cell counts normal for age  CMV negative  EBV negative  Parvovirus negative  Gaucher’s disease screening negative  Nieman Pick disease screening negative  Ferritin 1140 ng/dl | CRP +++  ESR 30-115 mm/hr  Ferritin 624 ng/dl  High C3, normal C4 and normal CH50  TORCH infectious studies: negative |

*Clinical documentation only without specific lab values recorded.

**Obtained during critical illness

^#^Patient on steroids
