# Supplementary material for: Biallelic loss-of-function PTPN6 variants lead to inflammatory lung disease and hemolytic anemia
Source: J Exp Med. 2026 Oct 7;223(11):e20252379. doi: 10.1084/jem.20252379 (PMC13644687; doi:10.1084/jem.20252379)
Supplement: Supplementary file 5 [file jem_20252379_tables2.docx]

**Table S2.** Peripheral blood cell count for patients C(II-2), D(II-2), and E(II-1) indicating anemia during infancy.

| **Patient** | **Age** | **Blood Leukocyte Count (x 10^9^/L)** | **Hemoglobin (g/dL)** | **Platelets**  **(x 10^9^/L)** | **Blood Neutrophil Count (x 109/L)** |
| --- | --- | --- | --- | --- | --- |
| **C(II-2)** | **2 months** | 5.3 (6-17) | 8.4 (10-18) | 89 (140-450) | 2 (0.78-7.7) |
|  | **3 months** | 7.6 (6-17) | 7.5 (9-14) | 140 (140-450) | 3.1 (0.78-7.7) |
|  | **15 months** | 2 (6-17) | 6.7 (10.5-13.5) | 66 (140-450) | 1.1 (0.9-7.82) |
|  | **16 months** | 0.3 (6-17) | 6.2 (10.5-13.5) | 40 (140-450) | 0.24 (0.9-7.82) |
|  |  |  |  |  |  |
| **D(II-2)** | **1 Year** | 17.6 (5.5-17.5) | 7.9 (11-13.5) | 609 (140-450) | 13.8 (1.0-8.5) |
|  | **8 Year** | 26.8 (4.5-15.5) | 12.9 (11.4-15.5) | 903 (140-450) | 20.95 (1.5-8.5) |
|  |  |  |  |  |  |
| **E(II-1)** | **1 week** | 9.2 (5-21) | 8 (13.5-19.5) | 44 (140-450) | 1.93 (0.95-13.44) |
|  | **2 weeks** | 7.1 (5-20) | 8.2 12.5-20.5) | 62 (140-450) | 0.81 (0.7-9.6) |
|  | **2 months** | 10.2 (6-17) | 6.7 (9-14) | 76 (140-450) | 1.84 (0.78-7.7) |
|  | **5 months** | 6.1 (6-17) | 4.9 (9.5-13.5) | 83 (140-450) | 3.05 (0.78-7.7) |
